# The Perception of Materials through Oral Sensation

**DOI:** 10.1371/journal.pone.0105035

**Published:** 2014-08-19

**Authors:** Philip D. Howes, Supinya Wongsriruksa, Zoe Laughlin, Harry J. Witchel, Mark Miodownik

**Affiliations:** 1 Materials Department, Imperial College London, London, United Kingdom; 2 Physics Department, King's College London, London, United Kingdom; 3 Institute of Making, University College London, London, United Kingdom; 4 Brighton and Sussex Medical School, University of Sussex Campus, Brighton, United Kingdom; University of Chicago, United States of America

## Abstract

This paper presents the results of a multimodal study of oral perception conducted with a set of material samples made from metals, polymers and woods, in which both the somatosensory and taste factors were examined. A multidimensional scaling analysis coupled with subjective attribute ratings was performed to assess these factors both qualitatively and quantitatively. The perceptual somatosensory factors of warmth, hardness and roughness dominated over the basic taste factors, and roughness was observed to be a less significant sensation compared to touch-only experiments. The perceptual somatosensory ratings were compared directly with physical property data in order to assess the correlation between the perceived properties and measured physical properties. In each case, a strong correlation was observed, suggesting that physical properties may be useful in industrial design for predicting oral perception.

## Introduction

Somatosensory sensations are known to contribute to the taste experience, with interactions taking place between gustatory and somatosensory stimuli at every level of the taste system, and chemical, thermal, and mechanical stimuli merging into coherent perceptions of foods and beverages [1]. However, it is not known how this sensory integration applies to the oral perception of solid materials, such as those used for eating and drinking.

Many psychophysical experiments have focused on understanding the fundamental perceptual factors which govern our haptic interface with the material world [2]–[6] (n.b. in this paper, when referring to ‘touch’ we are referring to contact between the fingers and a material, with reference to various works that have studied this). Such studies have revealed that the factors of roughness, warmth and hardness are amongst the most important sensations in assessment of surfaces through touch. In order to isolate and control variables, most of the work has involved looking at single perceptual variables. It is pertinent here to briefly review the definitions of these three dominant perceptual factors.

Surface roughness has been identified as the dominant factor in the exploration of surfaces by touch [7]–[9]. Surface roughness refers to height differences that occur in the profile of a surface, however perceptual roughness is more complicated, depending on various factors including the friction between surfaces, stickiness and pressure of touch [10]. The physical mechanisms involved in touch perception, and the way they combine and encode sensation, are very complex [11]. Various researchers have attempted to study the isolated mechanisms, such as vibration [12] and friction [10], [13]–[15], as well as the overall neural coding [16], [17]. The general consensus of these studies is that roughness perception is cognitively computed mainly through two distinct mechanisms. There is a vibration component for the detection of fine-structured surfaces, and a spatial variance component for gaining information from coarser surfaces [18].

The warmth of a material is another very distinct cue in the tactile exploration of its surface [4], [19]. The sensation of warmth is separate from the absolute temperature of a material; upon skin contact, what we perceive is the rate of heat transfer. Different materials transfer heat at different rates when they come into contact with the skin, and it is this reaction which allows us to identify the material [4]. The pertinent physical properties here are the thermal conductivity, heat capacity and density, which can be combined into a single variable called the thermal effusivity.

There are a number of physical properties that influence how hard we perceive something to be by touch. The elastic modulus is one of the more fundamental properties as it is independent of the dimensions of a material object. The elastic modulus is defined as the gradient of the stress–strain curve of a material in the elastic region (i.e. before it becomes permanently deformed). Materials with a high elastic modulus are usually stronger and stiffer, while those with a low elastic modulus are generally softer and more elastic. Previous studies suggest that the elastic modulus is the most important factor in the touch perception of harder materials, but for softer materials the stiffness is the dominant factor [20]. Therefore, if a material deforms a reasonable amount when pressed or squeezed, the stiffness correlates well with hardness perception. However, for materials which cannot easily be deformed, the elastic modulus correlates well with hardness perception [18], [20].

The sensation of metallic taste arising when certain solid metals or salt solutions are put in the mouth has been explored in depth [21]–[24]. Such studies have generally set out to investigate basic tastes, the argument being that as metallic taste does not fit into the traditional basic model, the model itself requires some sort of revision [25]. Recently Laughlin et al. conducted a study which looked exclusively at the taste of solid metals, with the objective of relating the perception of metallic taste with the physical properties of the metals [26]. A variety of spoons plated with various metals were used, with participants rating them on various sensoaesthetic attributes (metallic taste, hardness, sweetness etc.). They showed that the standard electrode potential, a measure of how easily atoms are oxidised, was a good predictor of metallic taste sensation. A follow-on study then investigated how the sensation experienced from the particular spoons affects the taste of flavoured creams, showing that zinc increases the perceived sweetness [27].

Many studies of perception use subjective rating scales to assess a participant's perception of a particular stimulus. Although this technique is ideal for studies that have a clear set of factors to be examined individually, studies assessing complex interactions of senses in which the nature and number of factors is unknown require a different approach since requesting participants to rate their experience of strictly defined factors may limit their response, or even suggest to them sensations that were not originally perceived. Furthermore, although the adjectives used in these scales are useful linguistic descriptions of experience, they may not be the best representation of the scientific nature of sensation [24], [25]. In this particular situation, techniques which avoid semantic biases may be employed to uncover the nature of sensorial experience in a more holistic fashion.

Multidimensional scaling (MDS) is a statistical modelling technique which allows the creation of visual maps of systems of objects which can have complex relationships [28], and has been used to great effect to uncover the dominant perceptual factors related to both touch [7]–[9] and taste [24]. For example, a study by Stevens *et al*. used MDS to reveal that ‘metallic’ tastes are distinctly separate from the basic tastes, and thus can be considered a taste category in its own right [24]. This visualisation of complex data is often useful in uncovering patterns which are not immediately obvious in the raw data or through more quantitative analysis techniques. MDS maps are based on comparisons between all objects in a group across multiple (and sometimes many) attributes, and as such does not rely on semantic labels. An MDS map consists of points which represent each object, where the proximity of the points indicates their similarity such that similar objects will be closer together and dissimilar objects farther apart. A dissimilarity matrix needs to be built as the input for MDS modelling, and this can be obtained through tasks such as similarity judgements of pairs, structured grouping or free sorting. In the context of MDS, the concept of ‘stress’ is used to quantify how distorted the data has to become to fit it into a given number of dimensions of perceptual space. High stress values indicate that there are likely more dimensions required to represent your data in a multidimensional space. Hence, it is useful look at a plot of stress versus dimensionality to identify how the stress changes with dimensionality.

By studying the dimensionality of the perceptual space it is possible to identify the number of factors which dominate the space. For example, if upon inspection of how stress changes with dimensionality it appears that three dimensions is the optimum solution for the data, this suggests that there are three dominant factors. However, from the dissimilarity data it is not possible to discern the identity of these factors, or which variables they relate to. To reveal this, additional information is needed. Subjective attribute ratings are often used for this, with the attribute ratings being compared with the MDS data in order to correlate the dimensions with the dominant factors. One method of doing this is linear regression. This is a useful tool for providing an objective interpretation of an MDS model, allowing the association of dimensions with specific factors [24], [28]. The subjective attribute ratings are regressed over the coordinates of the stimuli represented in the MDS model. This yields a set of vectors which indicate the directions through the MDS space which are maximally correlated with the ratings of each attribute. It is important to note that the results obtained in this process are dependent on the choice of attributes in the subjective ratings. The attributes that are chosen may or may not correspond directly to the criteria used by participants for their similarity judgments.

In general, studies of oral sensation with respect to tactile perception have only been conducted in the realm of dentistry and oral physiology [29]. Studies of oral stereognosis, the ability to recognise and discriminate the forms of objects when placed in the mouth, can be used as a test of oral health, for example in relation to patients under rehabilitation after serious oral or dental surgery [29]. The relationship between tongue sensation and tongue function in regards to speech, mastication and deglutition (swallowing) is of particular interest to rehabilitative professionals [30], [31]. Furthermore, as taste always occurs amid thermal and mechanical stimulation, the study of taste as a cutaneous sense has also been considered [1]. Engelen and Van Der Bilt have studied oral physiology and texture perception of semisolids in relation to how we perceive food as it is processed (chewed, diluted and broken down) in the oral cavity [32]. Their results implied that intra-individual differences in oral texture perception could be attributed to the variations in oral physiology (e.g. oral sensitivity, tongue movements, temperature and saliva composition). However, it is unknown to what extent oral physiology affects the perception of solid materials. Edmund Rolls has also studied various aspects of oral perception of semi-solid texture in the mouth, with particular emphasis on the somatosensory perception of fats and the neurological processing of complex multimodal oral stimuli [33].

The experiments reported in this paper were designed to study the multimodal perception of solid materials in the oral cavity, inclusive of taste, textural and thermal factors, amongst others. Our aims were three-fold: 1) to directly compare oral perception with previous touch-only perception studies, 2) to study the interaction between the taste and somatosensory modalities to establish which sensations are dominant for our stimuli set, and 3) to study the correspondence between the perception of warmth, hardness and roughness and a set of corresponding physical properties, which were thermal effusivity, elastic modulus and surface roughness, respectively. Although similar studies have been performed for the sense of touch through the skin, and fingers in particular, techniques of this kind have not been used to study oral sensation and perception before. The implications of this work reach beyond simply improving the understanding of oral perception. The idea of investigating how physical properties relate to psychophysical properties is linked to attempts to forge stronger links between materials science and design [34]. Furthermore, these ideas have an immediate application in the development of tactile branding and product identity of products associated with eating and oral use [35].

## Materials and Methods

### Ethical Statement

Ethical consent for the study was provided by the King's College London local ethical review board. Upon agreeing to take part in the study, all participants signed a consent form but were free to withdraw at any point.

### Participants

Thirty-eight participants (30 for experiment 1, and 8 for experiment 2) of mixed ages and sexes were recruited for the study. To participate, participants were required to be between 18 and 65 years of age, and in good general health. They were informed that if they were pregnant, suffering from a cold or flu, or afflicted by any general medical condition known to compromise the senses of taste and smell such as taste-based synaesthesia, any disorders of olfaction (anosmia, hyperosmia, hyposmia, dysosmia) and any disorders of taste (ageusia, dysgeusia), then they could not participate in the study. The upper age limit of 65 was set in an attempt to negate the effect of the loss of taste sensitivity during the normal ageing process [36]. No bias was given for or against anyone as a result of their gender, ethnicity or nationality.

### Apparatus

**Figure 1:**
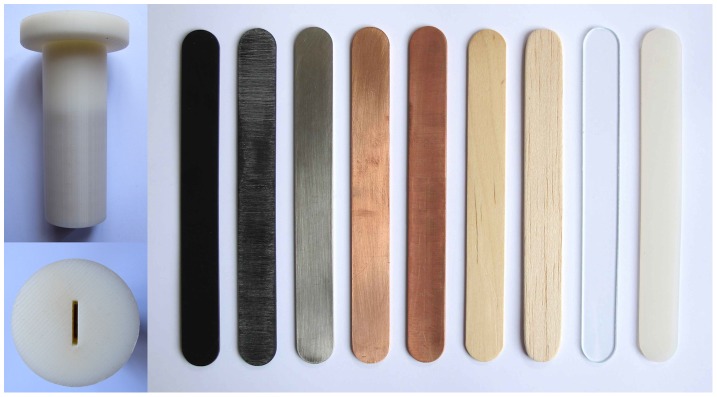
The nine stimuli used in the study, with one of the weighted ABS handles used to hold the stimuli during the experiment. From left to right: polystyrene (PS), rough polystyrene (R PS), stainless steel, copper, rough copper, birch, balsa, glass and silicone.

In the experiments, participants were asked to place material stimuli in their mouth. These stimuli were shaped like ‘lolly sticks’ (and referred to as such) in order to make the participants associate the samples with something which they will feel comfortable putting in their mouth (see Figure 1). The stimuli were 150 mm by 17 mm, and were cut with an aqua jet cutter (Aqua Dynamics Ltd, UK) from 2 mm thick sheets. Only non-toxic materials, suitable for culinary use, were used. There were 9 stimuli in total, made of birch wood, glass, balsa wood, stainless steel, silicone, two from copper and two from polystyrene plastic. Commercially available birch lolly sticks were purchased from Loypack (Poulton Le Fylde, UK), and used as the model on which the others were based. These were used as received. 2 mm thick sheets of all other materials were purchased and used as received. Two samples were created by grinding the materials with 60 grit silicon carbide paper (rough polystyrene and rough copper), followed by extensive washing to remove any traces of particulate material. All sticks were thoroughly washed, sterilised, and dried before use. The wooden, plastic and silicone sticks were disposable, and the others were thoroughly cleaned and sterilised for each participant.

The stimuli were presented to the participants in holders (handles) to stop them touching the surface and receiving tactile cues from their fingers. The handles were constructed from ABS plastic on a Dimension Elite 3D Rapid Prototyper. The holders were weighted to make them heavy, such that weight differences between the sticks were masked by the weight of the handle. These measures were designed to ensure that the participants were judging the objects from oral sensation alone.

### Experiment 1

This experiment was performed with 30 participants, and in two parts. Participants were seated at a table covered with a black table cloth, sat opposite the researcher conducting the experiment. All studies were performed during daylight hours, and sessions lasted 30 mins on average and no longer than 45 mins in any case. The participants were given a fresh bottle of water and encouraged to drink at regular intervals to refresh their palate. The participants were instructed that they could move the stimuli around in the mouth, but could not bite or bend them, and were asked to wear a blindfold. In the first part, data was generated for a paired-comparison MDS analysis. The participants were presented with a pair of stimuli. They were asked to put one in their mouth, and to focus on the sensations they experienced. This was repeated with the second stimulus. They were then asked to rate how similar the two stimuli were using a numbered rating scale of 0 to 20, where 0 indicated that the stimuli were completely dissimilar and 20 indicated that they were identical. A trial run of 5 pairs was conducted at the beginning to give the participants chance to adjust to the system of judgement, all of which were discounted from data analysis. A set of 9 sticks gives 45 possible pairs, including identical pairs but discounting repeated pairs. Each individual participant was asked to do 15 of these pairs. In total, there were 20 pairs of stimuli presented in 4 groups of 5, with short breaks in between. In order to minimise participant fatigue, these repetitions were kept deliberately low compared to similar literature studies. Collectively over the 30 participants, 10 assessments were collected for each stimulus pair, and the order of presentation was randomised. The mean value of these 10 assessments was calculated for each stimulus pair. These mean values were then used to create a dissimilarity matrix. The order of presentation within a pair was reversed for half of the participants in order to discount ordering effects.

In the second part of the experiment, the same 30 participants were asked to judge the stimuli one at a time. The whole set of 9 stimuli was used, with one repeat stimulus (steel) to be presented first and last to serve as measure of reliability, and as a discountable first data point. The other stimuli were presented in a random order. When the participants were assessing each stimulus they were asked to rate each one on a variety of subjective attribute Likert (1 to 7) scales, specifically warmth (0 not warm to 7 very warm), hardness (0 not hard to 7 very hard), roughness (0 not rough to 7 very rough), bitterness (0 not bitter to 7 very bitter), sweetness (0 not sweet to 7 very sweet), sourness (0 not sour to 7 very sour) and saltiness (0 not salty to 7 very salty).

### Experiment 2

In a second experiment, conducted separately and with different participants, 8 participants were asked to assess the stimuli one at a time. This experiment was designed to allow the participants to freely describe their sensations without prescribed rating scales. Whilst wearing a blindfold, the participants were handed the stimulus in its holder and then asked the following questions: “What is the dominant sensation?”, “How does the stick feel?” and “How does the stick taste?”. The responses were noted down by the investigator. This part of the experiment was not quantitative, but intended to study the type and range of descriptors used by the participants.

### Material Properties

The elastic modulus, thermal conductivity, heat capacity and density of all the material samples were obtained from the Cambridge Engineering Selector database [37]. Where a value range was given for any particular property, the median was taken and the error was calculated from the extent of the range. Surface roughness of all the samples was measured using a surface roughness tester (Dektak XT Profilometer), with a measurement length of 10 mm. The arithmetical average surface roughness (*R*_a_) was obtained directly from the device, which measures the arithmetic average of absolute values of the irregularities on the surface.

### Data Analysis

All data analysis was carried out with IBM SPSS Statistics 19 (IBM Corporation, New York, USA), and all data plots were produced on Origin 8.5.1 (OriginLab Corporation, Northampton, USA). A dissimilarity matrix was created from the data of the comparisons task in experiment 1. This was processed with the PROXSCAL MDS algorithm in SPSS, which minimises raw stress with dimension reduction. A one-step 3D linear regression was performed in SPSS, and the beta coefficients were taken from this analysis. A linear regression was performed in SPSS of the perceptual factor ratings gathered in experiment 1 over the MDS plot coordinates, with the R-squared and significance values being used to determine the strength of correlation. Significance values (*P* values) were studied at two levels; a <0.05 level to indicate a reasonable correlation, and a <0.001 level to indicate a strong correlation. We also performed a principal component analysis with a varimax rotation.

Repeated measures one-way analysis of variance (ANOVA) was performed on the perceptual factor ratings in part 2 of experiment 1, in order to ascertain which factors exhibited statistically significant variance across the stimuli set over the course of the 30 participants. Significance levels (*P* values) of <0.05 and <0.001 were used to judge the degree of variance across the set. The nonparametric Spearman's rank order test was used to test the strength of correlation between the perceptual factor ratings and the relevant physical variables, where a significance level of 0.05 was used as a significance threshold. The physical property data was plotted against the corresponding perceptual data for the warmth, hardness and roughness on logarithmic scales [38].

In order to judge the consistency of the participants' responses in the perceptual factor ratings task, the stainless steel was presented twice, as the first and last stimulus. Data from the first one was only used for this comparison and was discounted in other analyses. The responses for each perceptual factor were compared between the first and last stimulus using a repeated measures one-way ANOVA to assess whether there was a significant difference between the two presentations of the steel stimulus. None of the perceptual factors showed a statistically significant change (P>0.05), except roughness (F(1,29) = 4.67, P = 0.04). Although this result may suggest that there is a change in the perception of roughness as the experiment progressed, we lack further evidence to support this and consider it more likely that an anomalous result in the roughness judgement was enough to bring this into significance. Overall, we consider these tests to show that there was good repeatability of results across the stimuli set for the perceptual ratings task.

## Results

### MDS Study

**Figure 2:**
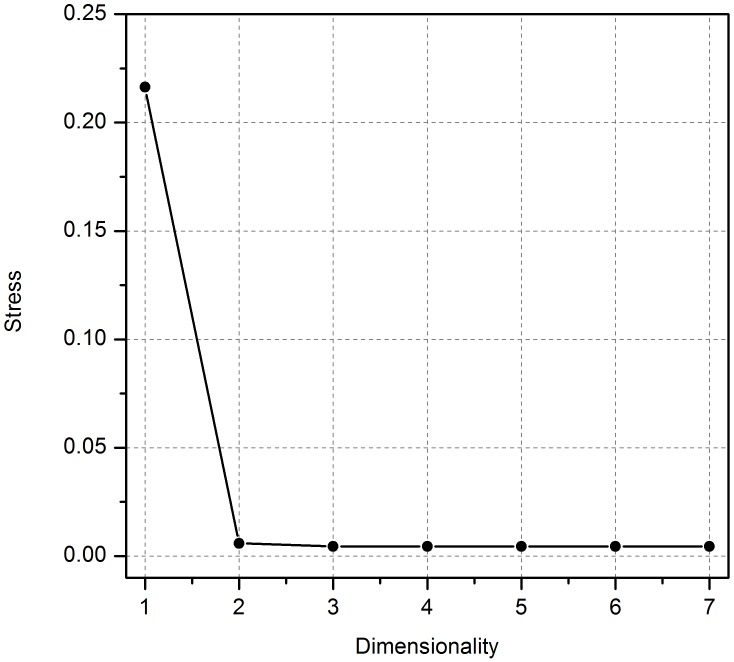
A scree plot showing the reduction normalised raw stress with an increase in dimensionality. A pronounced elbow at 2 dimensions suggests that the data may be most simply explained using a two dimensional MDS plot.

A scree plot of normalised raw stress against dimensionality was plotted, as shown in Figure 2. As the PROXSCAL MDS procedure runs, it attempts to minimise raw stress as dimensionality is reduced. The plot in Figure 2 exhibits a pronounced elbow at 2 dimensions. There is also a smaller drop in stress between dimensions 2 and 3 (of around 50%), before the change in stress levels off completely.

**Figure 3:**
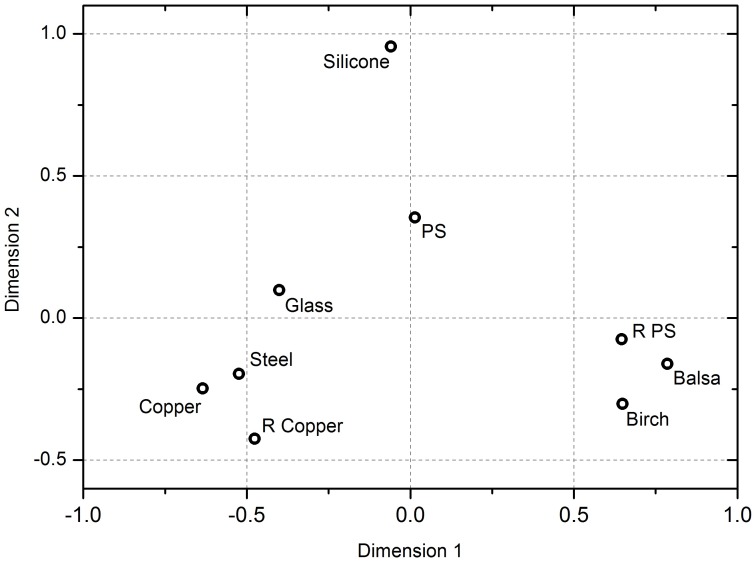
The MDS solution plotted in two-dimensions. The data positions show that the participants perceived similarities between the metals, and between the woods and rough polystyrene. The spacing between the glass, polystyrene and silicone suggest these were perceived as being dissimilar to any other of the materials. The isolation of silicone suggests it was perceived as being significantly different to all other materials.

Figure 2 suggests that the results may be most simply explained using a two dimensional MDS plot (three dimensions is a slightly better fit, but the extra dimension adds noise for very little congruence). Figure 3 shows the data as a 2D scatter plot. Two distinct groupings, and a number of outliers, can be identified. On the left hand side there is tight grouping of metals (copper, rough copper and steel). Glass plots near the metals group, but with a distinct separation. On the right hand side of the plot there is a tight grouping of the woods plus rough polystyrene. Polystyrene and silicone sit in isolation between these two groupings, with silicone plotting particularly far away from the other materials. These positions show that the participants found perceptual similarities between the metals, and between the woods and rough polystyrene. The spacing between the glass, polystyrene and silicone suggest these were not perceived as being significantly similar to any other of the materials, with the isolation of silicone suggesting that it was perceived as being significantly different to all other materials.

**Figure 4:**
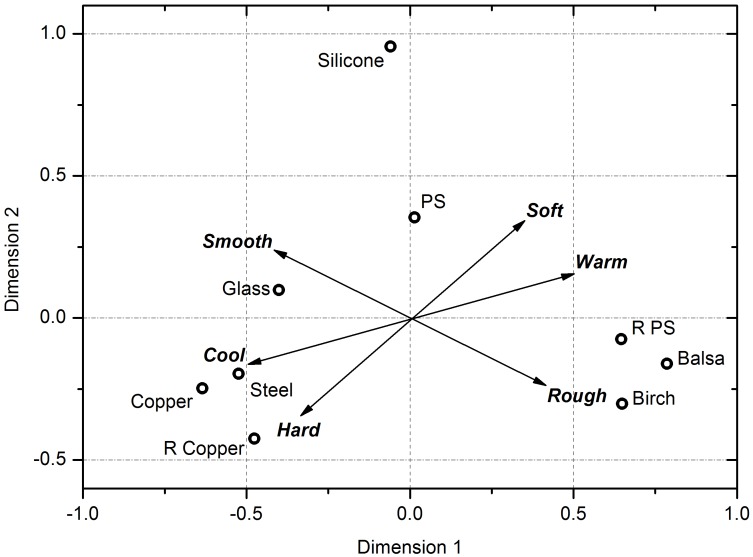
The two-dimensional MDS solutions plotted, with the subjective tactile ratings regressed over the MDS coordinates and plotted as vectors. Two tight groupings (metals in the bottom left, woods and rough polystyrene in the bottom right) are seen. Metals sit in a tight group between the hard and the cold vectors, whilst the woods and rough polystyrene sit between the rough and the warm vectors, revealing that the metals were perceived as ‘cold and hard’ and the woods and rough polystyrene perceived as ‘warm and rough’.

Using a stepwise regression method, vectors were derived from the somatosensory perceptual factor ratings of the participants that correlated with the data. The best fit, shown in Figure 4, was a model which used the combined perceptions of ‘warmth and roughness’ and ‘hardness and roughness’ (*R* ^2^ = 0.947, *P*< = 0.001). The position of the two tight groupings (metals in the bottom left, woods and rough polystyrene in the bottom right) in relation to the vectors tell us something of how these materials were perceived. The metals sit in a tight group between the hard and the cold vectors, whilst the woods and rough polystyrene sit between the rough and the warm vectors in Figure 4. This reveals that the metals grouped as they were perceived as ‘cold and hard’, whilst the woods and rough polystyrene grouped as they were perceived as ‘warm and rough’. We performed a principal component analysis which confirmed this analysis.

The small angle between the hard–soft line and the warm–cold line in the plots in Figure 4 suggests that these are not completely independent dimensions within this data set, and that there is a degree of correspondence between them i.e. the materials roughly break into classes of ‘warm and soft’ and ‘cold and hard’. None of the other adjectives were significant in the stepwise regression analysis.

**Figure 5:**
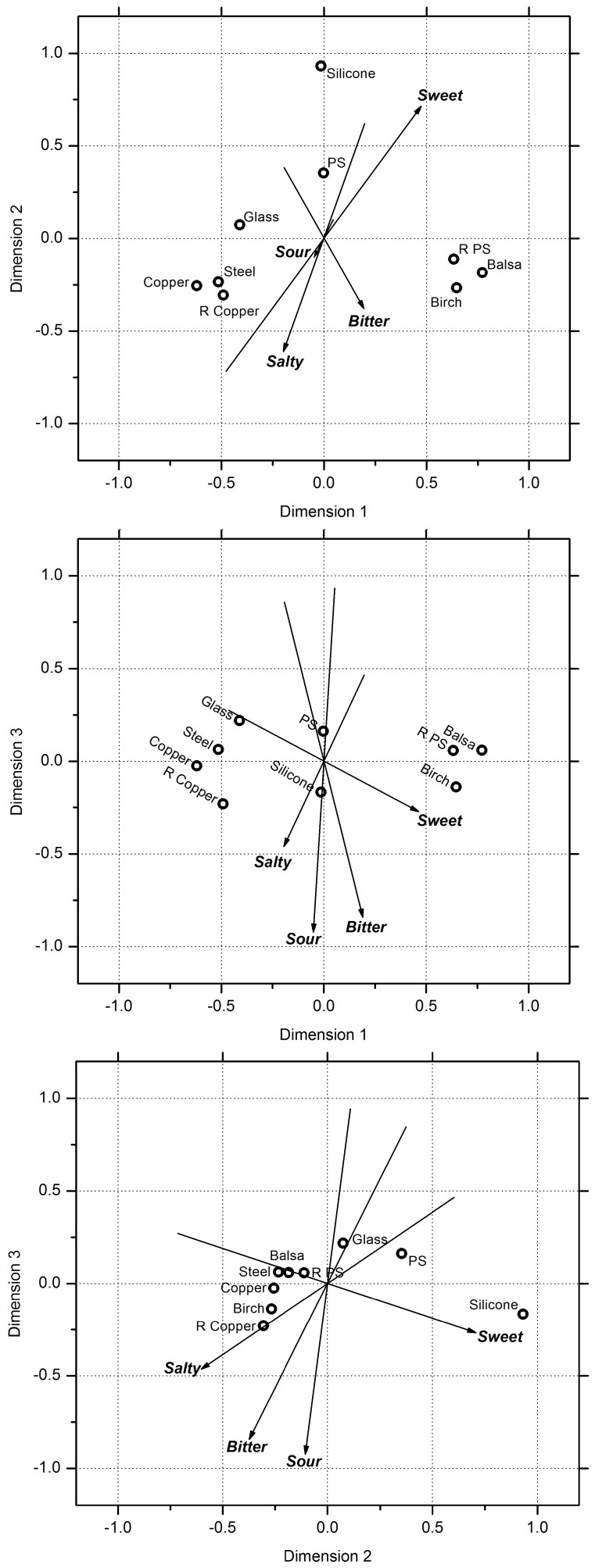
The three-dimensional MDS solutions plotted in paired dimensions, with the subjective taste ratings regressed over the MDS coordinates and plotted as vectors.

The taste factors (bitterness, sweetness, saltiness, sourness), were not dominant in the perception of these materials and are not needed to correlate the data in 2 dimensions. If we extend the analysis to a three dimensional MDS, the taste factors can discriminate between the materials to a minor extent: bitter (*R* ^2^ = 0.90, *P* = 0.0055), sour (*R* ^2^ = 0.89, *P* = 0.0070) and sweet (*R* ^2^ = 0.82, *P* = 0.026). Figure 5 shows the three-dimensional MDS solutions plotted in paired dimensions, with the subjective taste rating vectors. In plot A, it can be seen that there is a distinct directional difference between sweet and the other tastes (bitter, sour, salty). It is interesting to note that the general pattern of the taste vectors in Figure 5 is in the vertical direction, whereas the general direction of the tactile vectors in Figure 4 is in the horizontal direction.

Our data does not show a clear correspondence between the individual dimensions of the perceptual space and the adjectives which were rated by the participants. The fact that the somatosensory vectors are relatively planar in the first and second dimensions suggests that these dimensions are somatosensory based, whilst the taste vectors sit relatively orthogonal to the somatosensory vectors and spread into the third dimension, which suggests that this dimension was taste based. The stress reduction was relatively low going into the third dimension, which is another indicator that the somatosensory factors were dominant over the taste factors. We chose to use the basic tastes of bitter, sweet, sour and salty coupled with the dominant tactile factors revealed by touch-only studies, roughness, hardness and coldness [18]. However, it is likely that these adjectives did not wholly describe the oral perception, and that other more descriptive adjectives, for example woody or metallic tastes, or tactile slipperiness, may do.

In experiment 2, we asked eight new participants to describe the oral sensation while sampling the materials, which gave them freedom to describe the sensations in their own words rather than on prescribed scales. Although all of the adjectives that were used in experiment 1 were mentioned by the group (with the exception of salty), the responses were much more varied than could have been accounted for in the perceptual ratings task. In this work, we are considering only the basic tastes model, but it is important to acknowledge the role of flavour (inclusive of olfactory sensation) as well as taste. Given freedom to describe their sensations, the participants naturally moved away from basic tastes to use more descriptive language, citing flavours of various other materials. A summary of these responses in provided in Table 1. Some participants chose to describe the tastes in relation to foodstuffs (for example chestnut, popcorn and marzipan) which suggests that the basic tastes descriptors were not sufficient to encapsulate the sensations experienced. Some non-foodstuffs were also used as taste descriptors, for example soapy, bloody and earthy, which might be expected given that the samples were not edible. However, the variety of responses here was quite surprising, and indeed went far beyond the basic tastes model. It is interesting to note that only four of the chosen adjectives from experiment 1 were mentioned amongst the ‘dominant sensations’, and that these actually correspond to the four most highly correlated adjective vectors in the perceptual space (being hardness, warmth, roughness and bitterness), judging by the *R* ^2^ and significance values. This supports the result of the MDS test, which suggested that for the stimuli set used here, these four factors were the most appropriate in describing the participants' oral perception of the materials.

**Table 1:** A summary of the responses in Experiment 2, where the participants were given freedom to describe the sensations experienced when sampling the materials.

Taste	Somatosensory	Dominant Sensations
Earthy	*Smooth	Woody
Inert	*Hard	*Cool
Woody	Absorbent	*Smooth
Fibrous	Tough	Weird
*Bitter	Strong	Hilarious
Metallic	Fragile	Metallic
Burnt	Metallic	Slippery
*Sweet	*Rough	Rubbery
Savoury	*Cold	"Smell"
Soapy	*Warm	*Roughness
Chestnut	Slippery	Unpleasant
Popcorn	Sticky	Dry
Nothing	Rigid	Horrible
Bubblegum	Pulpy	*Bitterness
Marzipan	Solid	"Taste"
Pulpy	Synthetic	Synthetic
Bloody	Chalky	"Texture"
Chemical	Flimsy	
*Sour	Delicate	

*Factors which correspond to those which were tested in experiment 1.

### Perceptual Factors and Material Properties

Repeated measures one-way ANOVAs (with Greenhouse-Geisser correction) were conducted to ascertain which perceptual attributes varied significantly across the stimuli set. Saltiness showed no significant variation (F(3.85,111.57) = 1.53, *P* = 0.2), which suggests that this was not an important factor in the perceptual experience of the stimuli. However, sweetness (F(3.66,106.24) = 4.34, *P* = 0.0036) and sourness (F(3.64,105.44) = 2.94, *P* = 0.028) did show statistically significant variation to the 0.05 level, which shows us that there was a reasonable variation in the perception of these factors across the stimuli set. The warmth (F(4.67,135.29) = 87.28, *P*<0.0005), hardness (F(4.88,141.42) = 86.21, *P*<0.0005), roughness (F(3.51,101.93) = 109.89, *P*<0.0005) and bitterness (F(4.20,121.89) = 5.29, *P*<0.0005) all showed statistically significant variation to the 0.001 level, showing that the response of the participants varied significantly across the stimuli set for these four perceptual factors. This is in agreement with our observations in the MDS study.

**Figure 6:**
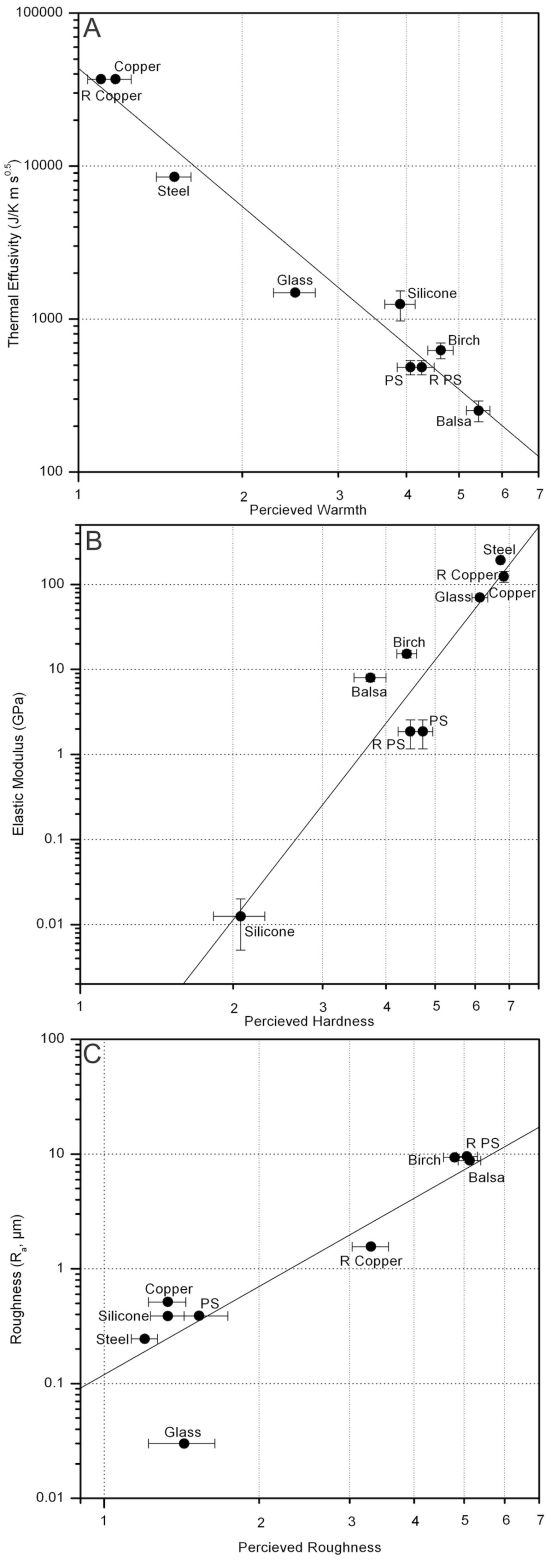
Log plots of the perceived warmth with thermal effusivity (*e*), perceived hardness with elastic modulus (*E*), and perceived roughness with surface roughness (*R*_a_), with the linear regression lines shown on each. A close correlation can be seen in all cases.

The perceptual factor ratings were used to ascertain how well the perceived qualities of the stimuli corresponded with the related physical properties data. The nonparametric Spearman's rank order test was used to analyse correlations between the perceptual factor ratings and the related physical properties. Specifically, we compared the perceived warmth with the thermal effusivity (*e*), the perceived hardness with the elastic modulus (*E*), and the perceived roughness with the surface roughness (*R*_a_). This showed that in each case, warmth (*ρ* = −0.94, *P* = 0.0002), hardness (*ρ* = 0.81, *P* = 0.008) and roughness (*ρ* = 0.80, *P* = 0.009), a strong correlation was present (see Figure 6).

The thermal effusivity was observed to offer a particularly close correspondence with its perceptual factor (warmth), as can be seen in Figure 6A. It is not surprising that this is the best correlation of the three tested as the thermal effusivity actually encapsulates three different physical properties (heat capacity, density and thermal conductivity), and as such acts to characterise the materials quite comprehensively. Furthermore, given that the mouth and tongue are necessarily highly sensitive to temperature, it would be expected that the participants' judgements of warmth would be sensitive to small changes in thermal material properties.

The elastic modulus showed a positive correlation with the perceived hardness (Figure 6B), although not as strongly as that observed with the thermal effusivity. In some ways this is surprising, given that the elastic modulus is a fundamental material property independent of sample dimension. From the result we can conclude that although this positive correlation does suggest a strong relationship between the elastic modulus and perceived hardness, there are likely to be other physical properties which influence the oral perception of hardness, stiffness for example.

The perceived roughness showed the least strong correlation with its physical property, in this case the measured surface roughness *R*_a_, however the correlation was still deemed to be strong. There was a close grouping of the stainless steel, copper, silicone, polystyrene and glass between 1 and 2 on the perceptual scale (as seen in Figure 6C), perceived as ‘smooth’ by the participants, despite their variation in measured surface roughness. This suggested that the participants could not differentiate accurately between samples of different roughness when the measured roughness was very low. In fact, from the plot it appears as if glass is an outlier, perceived as being of a similar roughness to the other ‘smooth’ materials but actually an order of magnitude lower as measured. Given the wet environment of the mouth, it is likely that the detection of the small scale ‘detail’ of textural variations is lowered as compared to that of the fingers due to a decrease in friction and important vibrational components [12].

Finally, it is pertinent to acknowledge the limitations of the current study. There are many factors (e.g. oral health, gender, age, time of day, how long since participants had eaten, what participants had eaten, whether participants were ‘supertasters’ or not) that were not addressed directly but could have had an impact upon the results obtained. Thus the results do not reveal any effects of these factors.

## Discussion

The results of our study have shown that the oral perception of solid materials can be represented in a multidimensional perceptual space akin to those used in separate touch [7] and taste [24] studies. In order to accurately predict the number of dimensions of the perceptual space for a given experiment a very large stimuli set is required, and this was beyond the scope of the present experiment. Given the limited number of stimuli used in this study we cannot exclude the existence of higher dimensions; however, we can say that there is likely to be no fewer than two. Indeed, there may be various higher dimensions related to more complex factors which stretch beyond the basic somatosensory and taste factors examined in this study. Our results from experiment 2, where the participants were free to describe their sensorial experience in their own words, elicited descriptive responses far beyond our basic factor set, and although these are very likely to be degenerate into a smaller set of factors, it seems likely that even this would stretch beyond our basic set. However, we did observe strong correlations between MDS data and a number of the sensorial factors tested, which has revealed that they are indeed relevant in oral perception, and we were thus able to address the specific aims of our study, as follows.

The first aim of our study was to directly compare oral perception with previous touch perception studies. In studies concerning touch only, typically using the fingers, the dominant factors have been identified as roughness, hardness, coldness and slipperiness [18], with roughness being the most significant in tactile perception. However, in our study roughness appears to be less important than the hardness and coldness, falling behind relative to tactile experiences. We suggest that this is because of the wet environment of the mouth lowering friction between the object and the skin [12], thus severely decreasing the vibrational component which is vital for roughness perception. This seems to have had the effect of ‘promoting’ the hardness and coldness in the order of perceptual importance relative to tactile studies investigating the fingers and skin.

Our second aim was to study the interaction between the taste and somatosensory modalities to establish which sensations are dominant for our stimuli set. From the MDS study, it was evident that the somatosensory perceptual factors dominated over the taste perceptual factors. The first two dimensions of the MDS seemed to account for most of the variability between the stimuli, and these dimensions were dominated by the somatosensory factors of warmth, hardness and roughness. However, it did not appear that there was a clear correlation between the factors and any particular dimensions. The weak third dimension seemed to relate to the taste factors, with bitterness rating as particularly relevant. However, movement into the third dimension was limited compared to the spread in the first two dimensions, which suggested that the taste factors were secondary to the somatosensory factors. Overall, we can say that, for this stimulus set, the main sensations used by the participants to distinguish between the stimuli were the warmth, hardness, roughness and to a lesser extent, bitterness.

Our third aim was to study the correspondence between the perception of warmth, hardness and roughness and a set of corresponding physical properties. The somatosensory perceptual factors all showed a strong correlation with their corresponding physical properties, suggesting that the use of materials data to predict tactile perception of materials may be extended to oral perception. The linear correlation was particularly striking for the thermal effusivity versus perceived warmth. These results demonstrate further evidence to that previously shown for another taste study [26], that there is a potentially rich body of quantitative data available from materials science databases that could be used to predict the perception of some psychophysical properties. It is hard to assess the impact of such an approach, although it seems likely that at the very least it would provide an inexpensive analytical tool for manufacturers of oral equipment, such as dental and medical apparatus, to identify promising materials. It may also be of use to artists, designers, chefs, and other makers and manufacturers of objects designed to go into the mouth, such as cups and cutlery.
